# Effects of non-severe acute kidney injury on clinical outcomes in critically ill patients

**DOI:** 10.1186/s13054-016-1295-4

**Published:** 2016-06-04

**Authors:** John A. Kellum, Raghavan Murugan

**Affiliations:** The Center for Critical Care Nephology, CRISMA (Clinical Research, Investigation, and Systems Modeling of Acute Illness) Center, Department of Critical Care Medicine, University of Pittsburgh School of Medicine, 3550 Terrace Street, Pittsburgh, PA 15261 USA

Acute kidney injury (AKI) is a serious medical condition estimated to affect more than ten million people around the world annually [1]. AKI results in a 1.7- to 6.9-fold increased risk of hospital mortality, and risk-adjusted rates of AKI and mortality appear similar across the world [2]. Patients who develop AKI also have worse kidney function at hospital discharge and thus far more risk for chronic kidney disease [2]. However, these risks are most clearly apparent for patients with very severe AKI, such as those who receive renal replacement therapy (RRT). Milder forms of AKI are less clearly associated with adverse outcomes and thus it is unclear whether mild AKI is in the causal pathway for morbidity and mortality. For example, in a large international study of patients cared for in the intensive care unit (ICU), stage 2 or 3 AKIs were strongly associated with mortality even after risk adjustment, whereas for patients incurring only stage 1 AKI the association was attenuated and no longer significant (odds ratio 1.68, 95 % confidence interval (CI) 0.89–3.17; *P* = 0.11) [2].

For purposes of this review, we define “non-severe AKI” as AKI that is not managed by RRT. We acknowledge that this is an imperfect definition because the decision to commence RRT is often a difficult one and there exists considerable heterogeneity across centers and among physicians, even experts. Thus, the same patient could be counted as non-severe if cared for by one clinician but severe if cared for by another. Although differences exist at the individual patient-provider level, rates of RRT for critically ill patients with AKI in general, though increasing, are relatively consistent around the world [2, 3]. For example, a decade ago, Uchino and colleagues found that in 23 countries on four continents 4.2 % of patients admitted to the ICU received RRT for AKI and this rate was not different between world regions (95 % CI 4.0–4.4 %) [3]. Similarly, Hoste and colleagues recently found that in 33 countries on five continents 13.5 % of all patients admitted to the ICU (excluding patients with end-stage renal disease) received RRT for AKI; again, the range was rather narrow (95 % CI 12.0–15.1 %) [2]. Approximately 25 % of critically ill patients with AKI receive RRT; this article is about the remaining 75 %.

## Acute kidney injury in less critically ill patients

A problem complementary to non-severe AKI is “AKI in non-severely ill patients”. One third of patients hospitalized for community-acquired pneumonia develop AKI and have significantly lower 1-year survival compared with those who do not develop AKI [4]. Furthermore, AKI is common even in patients outside the ICU or with non-severe pneumonia [4]. For example, we showed that AKI occurred in 25 % of patients admitted with community-acquired pneumonia to a hospital bed outside the ICU [4]. Despite the low overall acuity, patients developing AKI had a more than fourfold increased risk of death in-hospital (1.2 % versus 5.1 %) and a nearly twofold increase at 1 year (19.7 % versus 34.2 %; *P* < 0.001). Even within the ICU, patients with lower severity, defined by absence of circulatory or respiratory failure, had just as great an absolute risk (and higher relative risk) of death associated with AKI [5]. “Low-severity” patients may be at greater relative risk from AKI because of the perception that these patients are not severely ill. Similar results were observed by Joannidis and colleagues, who reported that AKI contributed more to mortality in patients with lower baseline severity of illness [6]. Such patients are usually judged to be at lower risk for developing AKI and are less likely to receive recommended interventions for high-risk patients such as avoidance of unnecessary nephrotoxic drugs and radiocontrast, close monitoring of serum creatinine and urine output, and assessment of fluid status [7]. The National Health Service in the UK has systematically assessed the care and outcomes for patients with AKI and found that inadequate risk assessment is common and is associated with delays in treatment, and investigators even judged some cases to have been avoidable altogether [8].

Similar limitations can be seen in studies evaluating novel biomarkers for AKI that have specifically focused on high-risk patients [9]. Ironically, although many more events will be seen in high-risk patients, the relative impact of AKI on survival is actually greatest in low-risk patients [5]. For patients without other organ failures, it may be easier, by virtue of their lower clinical complexity, to prevent AKI from developing in the first place. This leads to the supposition that efforts to curtail AKI in critically ill patients might be more effective if applied to low-risk patients or at least that low-risk patients should not be excluded.

## Limitations of current staging

In addition to the imprecision engendered by classifying AKI as “non-severe” by virtue of not being treated with RRT, the underlying AKI staging system first proposed by the Acute Dialysis Quality Initiative (ADQI) [10] and codified by the Kidney Disease Improving Global Outcomes (KDIGO) AKI workgroup [7] has important limitations. First, although urine output and serum creatinine are both measures of renal function and their changes do track, to some extent, with the severity of injury, the current staging system does not consider the effects of abnormalities in both domains compared with just one. For example, as shown in Table 1, the combination of oliguria and increases in serum creatinine portends far worse outcomes than either abnormality itself [11]. Thus, a patient with “stage 1” AKI by both serum creatinine and urine output criteria actually has the same risk of death by hospital discharge (11.3 %) than a patient with stage 2 criteria by serum creatinine alone (no oliguria) and a greater risk compared with stage 2 by urine output criteria alone (7.9 %). Similarly, adding oliguria (stage 1 criteria) to stage 3 creatinine criteria increases risk of death by more than threefold and risk for RRT by nearly fivefold. Adding creatinine (stage 1) to stage 3 urine output criteria has a twofold increased risk of death with a 10-fold increased risk for RRT. These short-term outcomes were mirrored by 1-year outcomes [11].

**Table 1:** Relationship between urine output and serum creatinine criteria and clinical outcomes

KDIGO stage		Urine output only				
		No AKI	Stage 1	Stage 2	Stage 3	Total
Serum creatinine only	No AKI	8179	3158	5421	440	17,198
	Dead	4.3 %	5.3 %	7.9 %	17.7 %	5.9 %
	RRT	0.0 %	0.0 %	0.1 %	1.1 %	0.1 %
	Stage 1	1889	1262	3485	842	7478
	Dead	8.0 %	11.3 %	13.0 %	32.1 %	13.6 %
	RRT	0.3 %	0.7 %	0.6 %	10.9 %	1.7 %
	Stage 2	618	476	1533	831	3458
	Dead	11.3 %	23.9 %	21.5 %	44.2 %	25.5 %
	RRT	1.0 %	1.3 %	1.7 %	21.7 %	6.3 %
	Stage 3	371	321	1,019	2,200	3911
	Dead	11.6 %	38.6 %	28.0 %	51.1 %	40.3 %
	RRT	3.2 %	17.8 %	14.2 %	55.3 %	36.6 %
	Total	11,057	5217	11,458	4313	32,045
	Dead	5.6 %	10.5 %	13.0 %	42.6 %	14.0 %
	RRT	0.3 %	1.4 %	1.7 %	34.6 %	5.6 %

*AKI* acute kidney injury, *KDIGO* Kidney Disease Improving Global Outcomes, *RRT* renal replacement therapy. Adapted from Kellum et al. [11] (with permission)

Another limitation to the KDIGO staging system is that duration of AKI is not considered [12]. Although even short episodes of AKI are associated with adverse outcomes [13], persistent AKI is clearly worse [11, 13, 14]. AKI may also appear less severe because serum creatinine is affected by disease severity. Creatinine is formed from non-enzymatic dehydration of creatine in the liver, and 98 % of the creatine pool is in muscle [15]. Thus, conditions which increase muscle breakdown may increase creatinine generation whereas conditions that reduce muscle mass, muscle perfusion, and liver function may decrease creatinine generation. Fluid overload may result in artificial lowering of the serum creatinine level and misclassification of AKI staging [16]. Finally, we should not forget that in patients with normal baseline renal function a clinical AKI event already represents substantial loss of kidney function. This is because the kidney has significant renal functional reserve such that more than 50 % of renal functional capacity must be lost before serum creatinine increases [17]. AKI biomarkers may be useful in closing this diagnostic gap. Some markers appear to be sensitive to “subclinical AKI” [18], whereas other markers appear to predict long-term outcomes, such as death or dialysis, only when patients develop clinical AKI [19].

For example, tissue inhibitor metalloproteinase-2 (TIMP-2) and insulin-like growth factor-binding protein-7 (IGFBP7) have been associated with 9-month incidence of a composite end point of all-cause mortality or the need for RRT in critically ill adults [19]. In univariate analysis, [TIMP-2]•[IGFBP7] of more than 2.0 was associated with increased risk of the composite end point (hazard ratio (HR) 2.11, 95 % CI 1.37–3.23; *P* < 0.001). In a multivariate analysis adjusted for the clinical model, [TIMP-2]•[IGFBP7] of more than 0.3 was associated with death or RRT only in subjects who developed AKI (compared with levels of not more than 0.3: HR 1.44, 95 % CI 1.00–2.06 for levels of more than 0.3 to 2.0, *P* = 0.05; and HR 2.16, 95 % CI 1.32–3.53 for levels of more than 2.0, *P* = 0.002) [19].

## Pathophysiology of non-severe acute kidney injury

If the associations between non-severe AKI and outcomes like mortality are causal, what would be the mechanisms? Even without being severe enough to warrant RRT, AKI affects multiple body systems. Three particularly important areas appear to be immune dysfunction, fluid overload, and adverse drug events (ADEs). Mehta and colleagues [20] reported that in 40 % of ICU patients with sepsis and AKI, sepsis occurred after AKI. This may in fact be an underestimate since they used serum creatinine—well known to be a delayed criterion—to diagnose AKI. Experimentally, we showed that AKI due to folic acid nephrotoxicity or myohemoglobinuria causes impaired neutrophil function apparent in vivo and in vitro [21]. Specifically, mice with AKI and pneumonia had worse bacterial invasion and lung injury than pneumonia alone, and neutrophils isolated from mice with AKI had impaired transmigration and F-actin polymerization in vitro. Together with an emerging literature on the various pathophysiologic effects of AKI [22], these results establish a scientific foundation for the effect of AKI on the immune system and on the risk for sepsis. They are also reminiscent of more well-established literature on the effects of chronic kidney disease on innate immunity.

Fluid overload has emerged as a major concern for clinically ill patients [23–25]. Fluid overload may lead to tissue edema that may affect virtually every organ system [26]. Cerebral edema may cause cognitive dysfunction. Pulmonary edema may lead to impaired gas exchange and prolong the need for mechanical ventilation. Myocardial edema leads to cardiac dysrhythmias and may impair myocardial performance. Edema of the liver, kidneys, and intestines can decrease organ function, and tissue edema can impair wound healing and predispose to skin breakdown. Ironically, fluid overload may actually be a larger problem for patients “not severe enough” for treatment with RRT since RRT is an effective way to manage fluid balance.

Finally, ADEs are common in the ICU, and AKI is a significant risk factor. Indeed, AKI shares a bidirectional relationship with ADEs. Drugs may complicate the course of as many as a quarter of patients with AKI in the ICU and up to 72 % in hospitalized patients [2, 3, 27]. Conversely, 770,000 hospitalized patients per year are injured or die because of ADEs [28], and AKI has been identified as a strong risk factor for ADEs [29]. A common cause of ADEs is the inappropriate prescribing of drugs, specifically in patients with AKI [30]. The majority of drugs are excreted by the kidney; so once AKI occurs, it is important to be vigilant to manage drug dosing and prevent resultant ADEs. Unfortunately, this is often not the case and drug reconciliation and dosing adjustment are often relegated to lower priority when the complexities of critical illness compete for clinician attention.

## Conclusions

Even when AKI is “less severe” and does not require RRT, it still may be in the causal pathway for morbidity and mortality in critically ill patients. Effects of renal dysfunction on immune function, fluid balance, and drug clearance may result in a myriad of complications, prolonging hospitalization and increasing risk of death. Recent advances in the understanding of AKI epidemiology, biomarkers, and electronic surveillance have offered potential solutions to the complex problems of identifying and managing AKI, but more is needed. Clinicians need to be aware of the risks and remain vigilant to prevent complications. Evolution in disease management from severe to “less severe” is familiar to us all. The emergence of non-ST segment myocardial infarction and “precancerous syndromes” are both examples of evolving patho-nomenclature as the epidemiology and pathobiology of diseases like coronary artery disease and cancer advance. We should expect the same as we move from acute renal failure to AKI.

## Abbreviations

ADE: adverse drug event
AKI: acute kidney injury
CI: confidence interval
HR: hazard ratio
ICU: intensive care unit
IGFBP7: insulin-like growth factor-binding protein 7
KDIGO: Kidney Disease Improving Global Outcomes
RRT: renal replacement therapy
TIMP-2: tissue inhibitor metalloproteinase-2
